# Hormonal contraceptive use and anaemia: a nation-wide pharmacoepidemiological study from Northern Europe

**DOI:** 10.1007/s10654-025-01272-3

**Published:** 2025-07-10

**Authors:** Sofie Ekroos, Elena Toffol, Oskari Heikinheimo, Jari Haukka, Mikko Arvas

**Affiliations:** 1 Faculty of Medicine, Medicum, University of Helsinki, Helsinki, Finland; 2 Research and Development, Finnish Red Cross Blood Service (FRCBS), Vantaa, Finland; 3 Department of Obstetrics and Gynaecology, University of Helsinki and Helsinki University Hospital, Helsinki, Finland

**Keywords:** Hormonal contraception, Iron deficiency anaemia, Women’s health, Pharmacoepidemiology

## Abstract

The WHO aims to reduce iron deficiency anaemia globally. Use of modern hormonal contraception (HC) could offer protection against anaemia in premenopausal women, but population-level effectiveness is unclear. We aim to quantify the effect of HC on anaemia. This nested case–control study includes over half the fertile-aged female population of Finland in 2017. Data on HC use from the national Prescription Center were combined with data on anaemia diagnosis from national care registries. Cases (anaemia diagnosis during follow-up, 2019–2020) were matched with up to five controls by age and municipality. After calculating the minimally sufficient adjustment set, adjusted odds ratios were derived in a conditional multivariable regression model accounting for matching. 3 100 cases of anaemia were matched with 13 143 controls. The minimally sufficient adjustment set included age, education level, obesity, abnormal uterine bleeding, and cancer. Compared to non-users of HC, risk of anaemia was lower in users of combined oral contraceptives containing ethinylestradiol (0·74 [0·66–0·83]) or oestradiol (0·49 [0·35–0·68]), progestin-only oral contraceptives (0·42 [0·35–0·51]), LNG-IUDs (0·64 [0·43–0·94]), and contraceptive vaginal rings (0·68 [0·49–0·94]). Individual product effects ranged from 0·77 [0·66–0·90] for drospirenone and ethinylestradiol to 0·40 [0·32–0·48] for desogestrel-only. Benefits of HC use extend to anaemia protection on population level. Anaemia protection should be included in guidelines on HC to support clinical decision making.

**Supplementary Information:**

The online version contains supplementary material available at 10.1007/s10654-025-01272-3.

## Introduction

Iron deficiency and subsequent iron deficiency anaemia (haemoglobin < 120 g/L, from here, anaemia) is a major contributor of global disease burden [1]. Reproductive aged women (15–49 years old) are at particular risk, with an estimated global prevalence of 30%. Accordingly, the World Health Organization (WHO) has set its reduction as a global health priority. Despite this, progress in reducing anaemia has stagnated. Therefore, a framework for action, “WHO Accelerating Anaemia Reduction”, was published in 2023 [2]. The framework brings together multiple sectors and actors, mapping out actions to improve the coverage of interventions. Interventions recommended include promoting voluntary use of modern hormonal contraceptives (HC) and medical management of gynaecological conditions, such as heavy menstrual bleeding (HMB). While approximately 0.87 mg of iron is lost during a normal menstrual cycle, women with HMB lose an estimated 5.2 mg of iron [3]. Despite a lifetime prevalence of up to 50%, HMB remains underdiagnosed [1].

In addition to reducing rates of unwanted pregnancy, short-acting (SARC; oral contraceptive pills, vaginal rings, and transdermal patches) and long-acting reversible contraceptives (LARC; intrauterine devices, and subdermal implants) have numerous health benefits, both from an individual and a public-health perspective [4]. HC, in particular levonorgestrel-releasing intrauterine devices (LNG-IUDs), reduce menstrual flow and are used as a treatment for anaemia in women with HMB [5]. Due to their suppressive and stabilizing effects on the endometrium, combined oral contraceptives (COCs) combining progestins with either ethinylestradiol or natural oestrogens are also widely used for this purpose [5, 6], with evidence of newer COCs with natural oestrogen offering a more favourable bleeding profile compared to ethinylestradiol containing COCs [7]. Systemic progestins are often recommended as a second-line treatment option for HMB due to their less favourable bleeding profile [5].

**Fig. 1:**
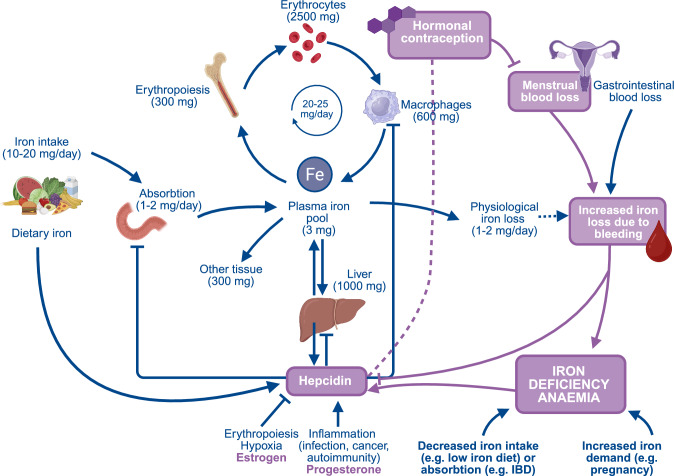
Schematic representation of possible mechanisms underlying how use of hormonal contraception may prevent development of anaemia. The diagram illustrates key pathways of iron metabolism, including intestinal absorption in the duodenum (1–2 mg/day), distribution across the body, daily turnover (20–25 mg/day) alongside with physiological iron loss (1–2 mg/day). Disruption in iron intake, pathological absorption, increased iron demand or bleeding can lead to anaemia. Hepcidin is produced by the liver and acts as the master regulator of iron homeostasis. Hepcidin is tightly modulated by multiple factors, including erythropoiesis, inflammation, sex hormones, physiologic and pathological bleeding, and anaemia. Hormonal contraception decreases menstrual blood loss and may modulate hepcidin through sex hormone response, leading to improved iron status. Figure created using Biorender

Different HCs vary in their reduction of menstrual blood loss. Additionally, they differ in suppression of ovarian function, leading to different levels of endogenous oestrogen and/or progesterone. Oestrogen correlates negatively and progesterone positively with serum hepcidin concentration. Hepcidin suppresses absorption of intestinal iron and red blood cell production [8]. Thus oestrogen-containing HC are likely to enhance iron absorption, whereas progestin-only HCs may decrease it. A schematic representation of the iron cycle, its relevance to anaemia and how use of HC may influence the development of anaemia is illustrated in Fig. 1.

Subsequently, different HC products are likely to affect iron balance in a product specific manner. Use of any HC was positively associated with haemoglobin levels in a Swedish prospective population study [9]. Other studies have identified oral contraceptives, contraceptive implants, and contraceptive injections to be protective against iron deficiency and anaemia at a population level in Tanzania [10, 11]. An analysis of 12 low- and middle-income countries found a pooled adjusted odds ratio of 0·68 (95% CI 0·64–0·73) for oral contraceptives. Similar results were found in a pooled analysis of 51 low- and middle-income countries, where HC users had lower odds (aOR 0·72; 95% CI 0·64–0·81) of anaemia [12]. Population-based studies grouped HC by route of administration, making it difficult to assess individual products. A literature review found evidence of LNG-IUDs reducing anaemia, but no studies on other HC types [4]. Conversely, an Indian study found higher odds of anaemia in oral contraceptive users, [13]. and multiple smaller studies from high-income regions on healthy women [14, 15] and blood donors [16, 17] did not associate systemic HC use to iron deficiency/anaemia.

While multiple studies link HC use to reduced menstrual blood loss, the effects of HC use on the risk of anaemia at a population level remain largely unknown. Moreover, even though studies have identified HC use to be protective against anaemia [9, 18], no large-scale epidemiological studies have assessed these effects of different HC. Using national registry data, we aimed to investigate the associations of modern HC and anaemia, and to quantify the effect in comparison to non-use of HC.

## Methods

### Study design

The current nationwide nested case–control (NCC) study is part of a large prospective register-based study on the risks and benefits of HC use in Finland, which has been described in detail previously [19]. Briefly, the original group of HC users included all fertile-aged (15–49 years old) women permanently residing in Finland with at least one redeemed prescription for HC in 2017. HC users (n = 294 445) were selected from the national Prescription Centre, which includes information on all prescribed and redeemed medications in Finland (also including HC from 2017) using the unique and permanent personal identification number given to persons at birth/immigration. A same-sized control group of women not using HC (no redeemed HC prescription in 2017) was formed utilizing data from the population register provided by Statistics Finland, matching by age and municipality of residence. Use of HC was defined as using the Anatomical Therapeutic Chemical (ATC) codes for G02B (contraceptives for topical use), G03A (hormonal contraceptives for systematic use), or G03HB (antiandrogens and oestrogens). As emergency contraceptives (G03AD) are widely available over the counter in Finland, 89 women with such a prescription and their matched individuals were excluded.

For the current study, cases were defined by identifying women diagnosed with anaemia during follow-up. Anaemia diagnoses were sought from two registries maintained by the Finnish Institute for Health and Welfare (THL). The first, the Register of Primary Health Care Visits (AvoHILMO), includes data on outpatient primary care visits (International Classification of Diseases, Tenth Revision [ICD-10] diagnosis codes and International Classification of Primary Care, 2nd edition [ICPC-2] care encounter codes). The second, the Care Register for Health Care (HILMO), includes data on visits to specialized outpatient care and hospitalizations (ICD-10 diagnosis codes).

Prevalent cases (i.e. a diagnosis of anaemia within five years prior to start of follow-up) (n = 11 527) and participants who died before start of follow-up (n = 228) were excluded. Follow-up started on 1 January 2019 and ended on 31 December 2020, making the maximum length of follow-up two years. Censoring occurred on end of follow-up (EOF), emigration, or death. The final cohort included 588 712 individuals (294 356 HC users and their matched reference group), representing 52% of fertile-aged women in Finland. HC use was followed-up through purchase data from the Prescription Center.

The study was approved by the Ethics Committee of the Faculty of Medicine, University of Helsinki (3/2018; March 29, 2018).

### Participants

**Fig. 2:**
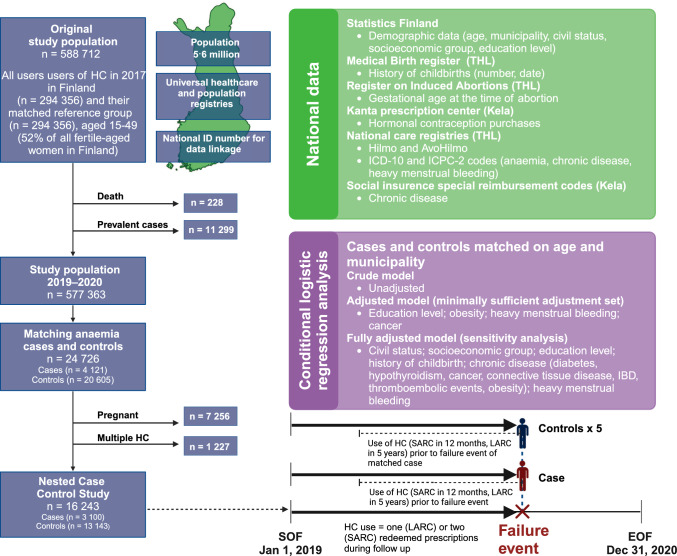
Flowchart illustrating the methodology of the nested case–control study investigating the incidence of iron deficiency anaemia among fertile-aged women residing in Finland in 2019–2020. Figure created with Biorender. Hilmo = Register of Primary Health Care visits. AvoHilmo = Care Register for Health Care. THL = Finnish Institute for Health and Welfare. Kela = Social Insurance Institution of Finland. ICD-10 = International Classification of Diseases, Tenth Revision. ICPC-2 = International Classification of Primary Care, 2nd edition. IBD = inflammatory bowel disease. Prevalent cases = diagnosis of anaemia within five years prior to follow-up. *SOF* start of follow-up, *EOF* end of follow-up. *HC* hormonal contraception. *SARC* short-acting reversible contraceptives. *LARC* long-acting reversible contraceptives

We constructed a NCC design aiming for five controls per case, matching by age (one-year range) and municipality to account for differences in regional prescribing patterns. Controls could be matched with multiple cases, but only once per case. Based on data from the Finnish National Medical Birth Register and the Register of Induced Abortions, cases and their matched controls, who were pregnant in the year prior to anaemia diagnosis (n = 7 256) were excluded. Additionally, women using multiple types of HC during the study period (n = 1 227) were excluded. An overview of the study design is depicted in Fig. 2.

As the study is based purely on registry data from electronic health records, obtaining individual level consent was unnecessary pursuant to the Act on the Secondary Use of Health and Social Data (26.4.2019/552) in Finland.

### Procedures

The outcome (failure event), anaemia, was classified as ICD-10 code D50 or ICPC-2 code B80, whichever occurred first during follow-up. Register data were combined using the personal identification number, which also indicated gender as recorded in the Population Register.

Based on data gathered from the Prescription Centre, SARC use was defined as having at least two consecutive redeemed prescriptions of the same ATC code in the year (360 days) prior to failure event. For LARC users (LNG-IUDs and contraceptive implants), only one redeemed prescription in the five years (1800 days) prior to failure event was required. For the logistic regression model of the NCC design, a categorical explanatory variable was constructed based on HC use in the past year. Additionally, a categorical variable for hormonal contraception classification was constructed (Table S1).

We received baseline variables from Statistics Finland, including the following as covariates for statistical model adjustments: civil status (unmarried, married, divorced, widowed, other), socio-economic group (self-employed, upper-level employees, lower-level employees, manual workers, students, pensioners, others, unknown), and highest level of education (upper secondary, post-secondary non-tertiary, short-cycle tertiary, bachelor, master, doctoral, unknown). Based on data from the Medical Birth Register, we added an indicator for history of childbirth (0; ≥ 1) and number of previous childbirths.

We added an indicator (no; yes) for chronic disease based on special reimbursement entitlement in one year before anaemia diagnosis (Social Insurance Institution of Finland, Table S1) for the following diagnoses: Diabetes Mellitus (type I and II), hypothyroidism, cancer, connective tissue disease, and inflammatory bowel disease. Furthermore, we created indicators for thrombosis and related events, obesity, and HMB in five years prior to anaemia diagnosis using ICD-10 codes from care registries.

### Statistical analysis

**Fig. 3:**
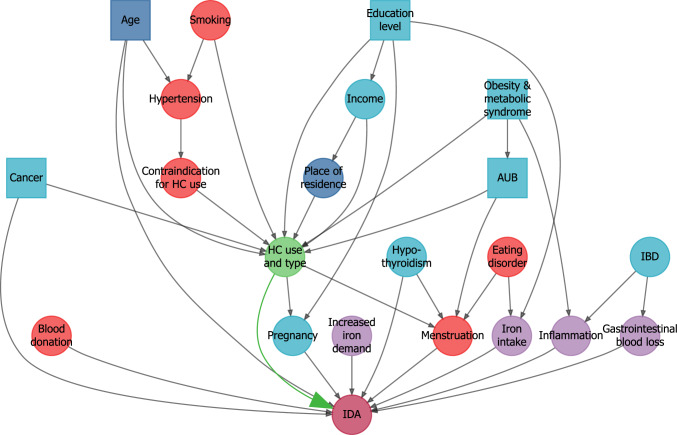
Directed acyclic graph (DAG) of major causal paths from literature used for model selection. Dark blue indicates matching criteria (age, municipality of residence), light blue represents variables used for model adjustment, red denotes unobserved variables, and purple signifies latent variables. Squares indicate the minimally sufficient adjustment set required. *AUB* abnormal uterine bleeding. *IBD* inflammatory bowel disease

For model selection (i.e. selection of confounders) for quantifying the effect of HC on anaemia, we first collected putative causal paths based on previous literature, including correlates of iron deficiency anaemia from FinRegistry, [20] and drew a mindmap of them (Figure S1). We then reduced the mindmap to a directed acylic graph (DAG) of unidirectional major causal paths and calculated the minimally sufficient adjustment set to be used in subsequent regression modelling (Fig. 3).

Baseline demographics were compared between cases and controls with chi-square test for categorical variables and one-way ANOVA/Kruskal–Wallis test, as appropriate, for continuous variables.

We used conditional logistic regression models, which accounted for matching in the NCC design. Results were reported as odds rations (OR) with 95% confidence intervals (95% CI). In addition to the crude and minimally sufficient adjusted models, we fitted a fully adjusted model accounting for civil status, socioeconomic group, highest level of education, history of childbirth, chronic disease (diabetes, hypothyroidism, cancer, connective tissue disease, inflammatory bowel disease, thrombosis and related events, and obesity), and HMB (Table S3, Panel A), to account for potential omitted-variable bias. Age was not included in the models due to matching on year of birth.

To address potential time-window bias, we fitted the models for two different time windows (three months (90 days; Table S3 Panel B), with a requirement of one redeemed prescription; six months (180 days; Table S3, Panel C), with the same requirement for redeemed prescription as described in the study design for the original models) of HC use.

Sensitivity analyses (Table S4) were performed to evaluate robustness of the models under the following conditions: adjusting for total number of previous childbirths instead of any history of childbirth (Model S1); excluding women under 25 years old to account for municipalities offering free HC to this age group (Model s2); and excluding disability pensioners to account for possible underlying pathology causing anaemia (Model s3).

Analyses were performed using R software version 4·0·5 for baseline demographics and NCC study (“tableone”, “Epi” and “survival” packages) and version 4·3·2 for DAG visualization and calculation of minimally sufficient adjustment (“ggdag” package).

### Role of the funding source

Funding was provided by The Finnish Red Cross Blood Service, Helsinki University Central Hospital, University of Helsinki, and The Biomedicum Helsinki Foundation. The funders had no role in the study design, data collection, data analysis, data interpretation, or writing of the report.

## Results

We identified 3 100 new cases of anaemia whom we matched with 13 143 controls. A total of 919 969 person-years were cumulated, with an overall incidence rate of anaemia of 3·37 (95% CI 3·25–3·49) per 1000 person-years.

Basic demographic characteristics and covariate information for cases and controls are presented in Table 1. HC use during follow-up is presented in Table 2. Cases were 0·69 times less likely to use HC (23·10%, 95% CI 21·62–24·62%) compared to controls, with a proportional difference of 10·31 percentage points (95% CI 7·98–12·60%). The age distribution was skewed in users of different HCs, with younger individuals more likely to use combined HC containing ethinylestradiol (Figure S2).

**Table 1:** Baseline characteristics of the nested case–control study population

	Overall	Cases	Controls	p
n	16243	3100	13143	
Age (mean (SD))	33**·**11 (9·49)	32**·**80 (9**·**14)	33**·**19 (9**·**57)	0**·**039
Number of previous childbirth (mean (SD))	0**·**81 (1**·**23)	0**·**85 (1**·**32)	0**·**80 (1**·**21)	0**·**075
Civil status, n (%)				** < 0·001**
Unmarried	10585 (65·2)	1914 (61·7)	8671 (66·0)	
Married	4506 (27·7)	951 (30·7)	3555 (27·0)	
Divorced	1093 (6·7)	221 (7·1)	872 (6·6)	
Widowed	35 (0·2)	7 (0·2)	28 (0·2)	
Other	24 (0·1)	7 (0·2)	17 (0·1)	
Socio-economic group, n (%)				** < 0·001**
Self-employed	614 (3·8)	107 (3·5)	507 (3·9)	
Upper-level employees	2095 (12·9)	373 (12·0)	1722 (13·1)	
Lower-level employees	5087 (31·3)	873 (28·2)	4214 (32·1)	
Manual workers	2290 (14·1)	386 (12·5)	1904 (14·5)	
Students	3348 (20·6)	660 (21·3)	2688 (20·5)	
Pensioners	360 (2·2)	105 (3·4)	255 (1·9)	
Others	1488 (9·2)	378 (12·2)	1110 (8·4)	
Unknown/NA	961 (5·9)	218 (7·0)	743 (5·7)	
Education, n (%)				** < 0·001**
Upper secondary	7223 (44·5)	1328 (42·8)	5895 (44·9)	
Post-secondary non-tertiary	117 (0·7)	15 (0·5)	102 (0·8)	
Short-cycle tertiary	539 (3·3)	81 (2·6)	458 (3·5)	
Bachelor	2936 (18·1)	546 (17·6)	2390 (18·2)	
Master	1764 (10·9)	334 (10·8)	1430 (10·9)	
Doctoral	104 (0·6)	18 (0·6)	86 (0·7)	
Unknown	3560 (21·9)	778 (25·1)	2782 (21·2)	
**Diabetes (Kela code 103 or 215), n (%)**	327 (2·0)	92 (3·0)	235 (1·8)	** < 0·001**
**Hypothyroidism (Kela code 104), n (%)**	153 (0·9)	52 (1·7)	101 (0·8)	** < 0·001**
**Breast cancer (Kela code 115), n (%)**	38 (0·2)	6 (0·2)	32 (0·2)	0·756
**Leukaemia, lymphoma, or other malignancy** **of blood or bone marrow (Kela code 117), n (%)**	19 (0·1)	< 5 (0·1)	16 (0·1)	0·941
**Gynaecologic cancer (Kela code 128), n (%)**	< 5 (0·0)	< 5 (0·0)	< 5 (0·0)	1·000
**Other malignancy (Kela code 130), n (%)**	14 (0·1)	< 5 (0·1)	11 (0·1)	1·000
**Connective tissue disease (Kela code 202), n (%)**	304 (1·9)	97 (3·1)	207 (1·6)	** < 0·001**
**Inflammatory bowel disease (Kela code 208), n (%)**	205 (1·3)	66 (2·1)	139 (1·1)	** < 0·001**
**Obesity (ICD-10 code E66), n (%)**	359 (2·2)	130 (4·2)	229 (1·7)	** < 0·001**
**Thrombosis, embolism or related endpoint** **event**^**a**^**, n (%)**	130 (0·8)	44 (1·4)	86 (0·7)	** < 0·001**
**Heavy menstrual bleeding**^**b**^**, n (%)**	909 (5·6)	323 (10·4)	586 (4·5)	** < 0·001**

Matching by age and municipality of residence. ^a^Diagnosis of thromboembolic event prior to start of follow-up based on ICD-10 codes (I80, I81, I82, I21, I26, I63). ^b^Diagnosis of heavy menstrual bleeding or related condition prior to start of follow-up based on ICD-10 codes (N80, N84, N85, N92, N93, D25)

**Table 2:** Use of hormonal contraception during follow-up among cases and controls

	Overall	Cases	Controls	p
**REFERENCE GROUP: no use of hormonal contraception**	11136 (68·6)	2384 (76·9)	8752 (66·6)	< 0·001
**Any hormonal contraception use**	5107 (31·4)	716 (23.1)	4391 (33.4)	< 0·001
**GROUP: combined oral contraceptives, EE**	2702 (16·6)	438 (14·1)	2264 (17·2)	< 0·001
Levonorgestrel and ethinylestradiol, fixed preparations	67 (0·4)	8 (0·3)	59 (0·4)	
Desogestrel and ethinylestradiol, fixed preparations	328 (2·0)	62 (2·0)	266 (2·0)	
Gestodene and ethinylestradiol, fixed preparations	555 (3·4)	65 (2·1)	490 (3·7)	
Drospirenone and ethinylestradiol, fixed preparations	1279 (7·9)	212 (6·8)	1067 (8·1)	
Dienogest and ethinylestradiol, fixed preparations	104 (0·6)	19 (0·6)	85 (0·6)	
Levonorgestrel and ethinylestradiol, sequential preparations	< 5 (0·0)	< 5 (0·0)	< 5 (0·0)	
Cyproterone and ethinylestradiol, fixed preparations*	366 (2·3)	71 (2·3)	295 (2·2)	
**GROUP: combined oral contraceptives, E2**	376 (2·3)	41 (1·3)	335 (2·5)	< 0·001
Nomegestrol and oestradiol, fixed preparations	178 (1·1)	18 (0·6)	160 (1·2)	
Dienogest and oestradiol, sequential preparations	198 (1·2)	23 (0·7)	175 (1·3)	
**GROUP: progestin-only oral contraceptives**	1332 (8·2)	130 (4·2)	1202 (9·1)	< 0·001
Norethisterone	101 (0·6)	15 (0·5)	86 (0·7)	
Desogestrel	1217 (7·5)	111 (3·6)	1106 (8·4)	
Drospirenone	14 (0·1)	< 5 (0·1)	10 (0·1)	
**GROUP: hormonal intrauterine device**; Plastic IUD with levonorgestrel	219 (1·3)	32 (1·0)	187 (1·4)	< 0·001
**GROUP: vaginal ring**; Etonogestrel and ethinylestradiol	312 (1·9)	48 (1·5)	264 (2·0)	< 0·001
**GROUP: contraceptive patch**; Norelgestromin and ethinylestradiol	41 (0·3)	8 (0·3)	33 (0·3)	< 0·001
**GROUP: contraceptive implant**	125 (0·8)	19 (0·6)	106 (0·8)	< 0·001
Etonogestrel	51 (0·3)	10 (0·3)	41 (0·3)	
Levonorgestrel	74 (0·5)	9 (0·3)	65 (0·5)	

IUD = intrauterine device. *Due to the classification of ATC code G03HB01 (cyproterone and oestrogens), the combined oral contraceptives with cyproterone and ethinylestradiol includes around 14% products in which cyproterone was combined with natural oestradiol, not ethinylestradiol

Based on DAG results (Fig. 2), the minimally sufficient adjustment set required to estimate the effect of HC use on anaemia was age, education level, obesity, abnormal uterine bleeding, and cancer.

**Fig. 4:**
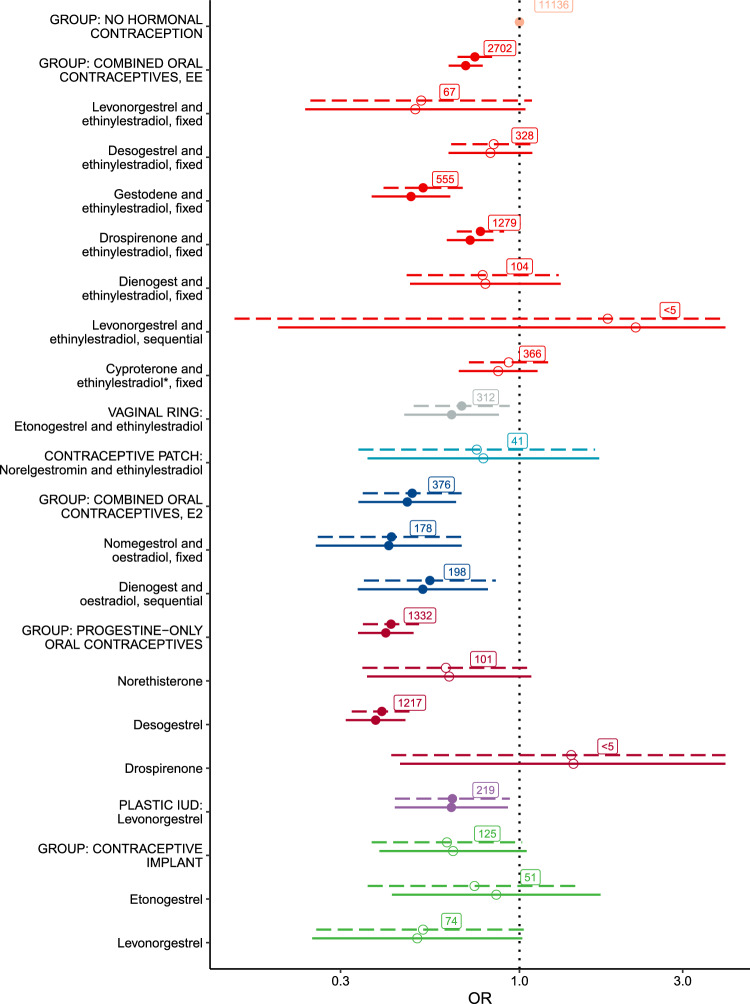
Risk of anaemia during use of different types of hormonal contraceptives. Conditional logistic regression results of the nested case–control study, taking into account matching (age; municipality of residence). The horizontal bars (solid for the crude model, dashed for the adjusted model) represent 95% confidence intervals of estimates of standardized coefficients (cut off for products levonorgestrel and ethinylestradiol, sequential [crude model 0·20–24·25, adjusted model 0·15–22·31] and drospirenone-only [crude model 0·45–4·62, adjusted model 0·42–4·75]). The circles show the distribution means. Filled circles highlight variables which confidence intervals contain the population mean with at least 95% confidence. Number of cases and controls using the specified product shown in square labels. The adjusted model accounted for education level, obesity, cancer, and heavy menstrual bleeding. *Due to the classification of ATC code G03HB01 (cyproterone and oestrogens), the combined oral contraceptives with cyproterone and ethinylestradiol includes around 14% products in which cyproterone was combined with natural oestradiol, not ethinylestradiol

Each contraceptive preparation was analysed separately in the NCC study (Fig. 4). Compared to non-users, risk of anaemia was significantly lower in users of ethinylestradiol containing COCs (aOR 0·74, 95% CI 0·66–0·83), oestradiol containing COCs (aOR 0·49, 95% CI 0·35–0·68), progestin-only contractive pills (POP) (aOR 0.42, 95% CI 0·35–0·51), LNG-IUDs (aOR 0·64, 95% CI 0·43–0·94), and vaginal rings (aOR 0·68, 95% CI 0·49–0·94).

According to odds ratios (Fig. 4 and Table S2), users of COC containing gestodene and ethinylestradiol had 48% lower risk of anaemia compared to non-users of HC (95% CI 32–60%), followed by nomegestrol and oestradiol (58% lower risk, 95% CI 31–74%), dienogest and oestradiol (45% lower risk, 95% CI 15–65%), and drospirenone and ethinylestradiol (23% lower risk, 95% CI 10–34%). Use of vaginal ring releasing desogestrel and ethinylestradiol was associated with a 32% lower risk (95% CI 6–51%), while desogestrel-only had the lowest risk (60%, 95% CI 52–68%).

The addition of covariates related to socioeconomic background, previous childbirth, and chronic disease in the fully adjusted supplemental model (Table S3, Panel A) did not cause substantial changes compared to the crude or adjusted models. To ensure that results were valid for different time windows, we changed the time window for HC use from 12 months to three and six months (Table S3, Panels B and C), with no notable difference in effect size estimates when accounting for the total number of users. Furthermore, adjusting for the number of previous childbirths, or excluding women younger than 25 years old, or excluding pensioners (Table S4), did not notably change results.

## Discussion

We found that HC users, both SARC and LARC types, have a significantly lower risk of anaemia compared to non-users of HC, ranging from 23% lower risk for users of oral contraceptives containing drospirenone and ethinylestradiol to 60% lower risk for users of desogestrel-only. By quantifying effect sizes, we demonstrate for the first time that on a population level, different HC products associate differently with iron balance in a product-specific manner.

Most of COCs, both those containing ethinylestradiol and natural oestradiol, were negatively associated with anaemia. Compared to COCs containing ethinylestradiol, natural oestradiol-containing COCs are associated with lower uterine bleeding, and some (dienogest and oestradiol) indicated for treatment of HMB [7]. Therefore, they are likely to offer better anaemia protection, as also supported by the present results. Notably, the effect size estimates for the natural oestrogen products were similar despite the difference in age profile of their users (Figure S2). This indicates that the effect is primarily attributable to the contraceptive itself, rather than any underlying variables. The lower risk of anaemia was not limited to combined oral contraceptives, but was also associated with the use of contraceptive vaginal rings.

Curiously, use of POCs, namely desogestrel-only was associated with the largest difference in effect size estimate compared to non-users, suggesting they offer the best protection against anaemia. This is somewhat surprising, as in contrast to combined oral contraceptives, POCs are associated with irregular bleeding, including amenorrhea and spotting. This suggests that, if well tolerated, their use does not need to be discontinued. Self-selection of long-time users with acceptable bleeding patterns or amenorrhea within this group is likewise possible, as women experiencing spotting throughout their cycle may decide to change HC type. However, single purchase of desogestrel POCs (signalling discontinuation of use) was not more common compared to other HC in the study population.

Interestingly, the use of LNG-IUD was associated only with a modestly (36%) reduced risk of anaemia compared to non-users, despite previous studies identifying LNG-IUDs as the most effective HC product in iron deficiency and anaemia prevention in healthy women [4, 17], as well as being used as first-line treatment for HMB. Our study is likely to underreport LNG-IUD use due to how Finland’s healthcare system is organized. Women using LNG-IUDs for contraception receive them often via prescription, while those with abnormal uterine bleeding receive the LNG-IUD used for therapeutic purposes directly from clinics. Moreover, starting from the mid-2010s, an increasing number of municipalities offer the first LARC free-of-charge, thus excluding these women from the study. Other LARCs (i.e. levonorgestrel and etonogestrel releasing implants) did not associate significantly with the risk of anaemia, likely due to a low number of implant users.

Our results contrast to previous, smaller studies on healthy women conducted in Italy and New Zealand [14, 15], as well as studies on Danish and Dutch whole blood donors [16, 17, 21], which found no associations between systemic HC use and iron deficiency and/or anaemia. However, our results complement previous population-level studies from low- and middle-income countries [10–12, 22, 23] by showing that the protective effect extends to populations in affluent countries. This was seen despite the likely differences in prescribing patterns and causes for anaemia. Additionally, our results suggest product-level differences.

It may be argued that the large number of users explain our significant results, as numerous users of a specific contraceptive preparation could act as a proxy for an established user base (i.e., women who have been using the product for multiple years without subjective side-effects and acceptable bleeding pattern), leading to lower odds of anaemia. Newer products, with a lower number and less established users, are more likely to be prescribed to younger women as their first HC, as opposed to women already satisfied with their HC. However, as the 95% CIs of the desogestrel-only POC, and COC containing drospirenone and ethinylestradiol, the two largest groups in comparison, do not overlap, we can be confident that their effect sizes actually differ, making this an unlikely sole explanation.

Decrease in volume of menstrual blood loss is likely the main mechanism for the decreased risk of anaemia in HC users. However, as there are differences even among the different groups, as well as the similarity in effect between fixed and sequential products, it is likely that some of the effect is caused by other mediating factors related to the hormone content. Additionally, some of the effect could be attributed to oestrogen-induced suppression of hepcidin, leading to higher iron absorption and reserves. Although previous studies have found that even short-term use of HC reduces the likelihood of anaemia, long-term use has been observed to further lower the odds. This suggests a cumulative effect of gradually improving iron stores [10, 12].

Despite our efforts to adjust for confounders, we recognise the limitations of this study. First, our study can only account for the HC purchases, excluding information on women who receive HC free-of-charge as part of municipal programmes available in around 70% of the 100 most populated municipalities in 2019. To account for this, we performed subgroup analysis excluding women less than 25 years-of-age, the age group most often included in these contraceptive programmes, which did not notably change results. Similarly, our study did not include data on anaemia diagnosis of women who only utilize private or occupational health care services. However, as results were similar in the crude model and the fully adjusted model, which accounted for socioeconomic group, it could be argued this loss of data decreases power, rather than precision. Second, a purchased HC product is not a guarantee of use. However, by requiring at least two subsequently redeemed prescriptions of the same product in one year window, we were able to control for primary non-compliance as well as minimise the risk of discontinuation of the prescribed HC. Thirdly, residual confounding due to unmeasured factors, such as genetic variance, smoking, diet, or iron supplementation, cannot be ruled out. The Finnish population is relatively genetically homogeneous, which limits the impact of variation in our cohort. By removing prevalent cases before start of follow-up we attempt to minimize residual confounding due to underlying predisposition to anaemia that could be caused by unmeasured risk factors. Prior diagnosis can be used as a proxy for underlying risk, as a clinically significant hereditary predisposition would likely have already manifested by adult age. Nevertheless, further studies with more ethnically diverse populations are warranted to examine confounding caused by varied genetic predispositions and lifestyle factors to anaemia.

Healthy-user bias could further confound results, as it is possible that health-conscious women seek healthcare more often and are more likely to be prescribed HC [24]. Women prescribed HC are also more likely to attend other healthcare providers. As such, estimates may be underestimated. In addition, the loss of information on therapeutic use of LNG-IUDs is likely to result these women being classified as non-users of HC, which likely causes an underestimation of the overall effect sizes.

In addition to revealing sources of bias for transparency, the directed acyclic graph aids in understanding causality of the measured associations. In theory, if our DAG correctly defines the paths between the exposure and outcome, observed associations shown in Fig. 3 would be estimates of causal effects. However, remaining bias depends on non-included biasing paths, such as bias introduced by missing information of LNG-IUDs and unmeasured confounders, including smoking and blood donation [25].

To the best of our knowledge, the present study is the first to assess the associations between hormonal contraceptive use and anaemia on a nationwide scale. A key strength of our study was the use of Finnish national registry data, allowing for a highly representative sample including over half of the fertile-aged female population of Finland. This allowed for a study design with high statistical power, that also accounts for possible regional differences in prescribing patterns.

Use of HC have been associated with increased risk of breast cancer and thrombosis, risks that should be clearly communicated to patients to support informed decision-making based on individual risk that considers genetic factors, age and lifestyle choices [26, 27]. Potential adverse effects must be evaluated in the context of the benefits HC offer, including improved quality of life in women suffering heavy or painful menstruation, decreased risk of ovarian cancer, and reproductive autonomy as well as reduced maternal mortality. Our results add to the existing knowledge on health effects of hormonal contraception use and expand knowledge on prevention of iron deficiency anaemia as set out by the World Health Organization’s framework for accelerating anaemia reduction. Furthermore, results can help decision-makers when designing family planning programmes, reimbursement rights of hormonal contraception, and national care guidelines. They are also of value at grassroots level by physicians in primary care settings and specialist gynaecological practices to support clinical decision making.

In conclusion, use of hormonal contraception is related to reduced odds of anaemia in women of reproductive age. The benefits of hormonal contraception extend beyond that of contraception and should be considered by policy-makers to promote women’s health and gender equity.

## Supplementary Information

Below is the link to the electronic supplementary material.

Supplementary file1 (PDF 664 KB)

## Data Availability

All codes for this study are available in GitHub. The deidentified data for running the analysis code is available from FinData (https://www.findata.fi/), with permission granted by the above mentioned.
