# Supplementary material for: Hormonal contraceptive use and anaemia: a nation-wide pharmacoepidemiological study from Northern Europe
Source: Eur J Epidemiol. 2025 Jul 10;40(8):925–35. doi: 10.1007/s10654-025-01272-3 (PMC12374858; doi:10.1007/s10654-025-01272-3)
Supplement: Supplementary file 1 — Supplementary file1 (PDF 664 KB) [file 10654_2025_1272_MOESM1_ESM.pdf]

Supplementary Materials for "Hormonal contraceptive use and anaemia: A nation-wide pharmacoepidemiological study from Northern Europe"

Sofie Ekroos, Elena Toffol, Oskari Heikinheimo, Jari Haukka, Mikko Arvas

Table of contents

Table S1 1

Table S2 9

Figure S1 10

Figure S2 11

Table S3 12

Table S4 13

Table S1: Overview of the definitions, units, and other specifications for outcome, exposure and each covariate used in the analyses. When applicable, the level used as comparison level has been bolded. ICD-10 = International Classification of Diseases, Tenth Revision; ICPC-2 = International Classification of Primary Care, 2nd edition; NA = not applicable.

Variable	Use in models	Definition	Unit/levels	Register and other specifics
Failure event	Outcome	Diagnosis of iron deficiency anaemia during follow-up based on ICD-10 code D50 or ICPC-2 code B80	TRUE; FALSE	Care Register of Primary Health Care Visits (Finnish Institute for Health and Welfare) and Care Register for Health Care (Finnish Institute for Health and Welfare)
Age	Matching criteria	Age at time of failure event (case) or at the time of failure event of matched case (control).	Years	Population Register (Statistics Finland)
Municipality of residence	Matching criteria	In this study, we refer to Sub-regional units as municipality of residence. Sub-regional units, a primary regional classification used in Finland's statistical system based on the administrative regional division, are area entities formed by one or more municipalities. Municipalities can differ in programmes	Helsinki [011]; Raasepori [014]; Porvoo [015]; Loviisa [016]; Åboland-Turunmaa [021]; Salo [022]; Turku [023]; Vakka-Suomi [024]; Loimaa [025]; Rauma [041]; Pori [043]; Pohjois-Satakunta [044]; Hämeenlinna	Population Register (Statistics Finland)

		offered to its citizens, for example some municipalities offer free-of-charge hormonal contraception to women under the age of 25 years.	[051]; Riihimäki [052]; Forssa [053]; Luoteis-Pirkanmaa [061]; Etelä-Pirkanmaa [063]; Tampere [064]; Lounais-Pirkanmaa [068]; Ylä-Pirkanmaa [069]; Lahti [071]; Kouvola [081]; Kotka-Hamina [082]; Lappeenranta [091]; Imatra [093]; Mikkeli [101]; Savonlinna [103]; Pieksämäki [105]; Ylä-Savo [111]; Kuopio [112]; Koillis-Savo [113]; Varkaus [114]; Sisä-Savo [115]; Joensuu [122]; Keski-Karjala [124]; Pielisen Karjala [125]; Jyväskylä [131]; Joutsa [132]; Keuruu [133]; Jämsä [134]; Äänekoski [135]; Saarijärvi-Viitasaari [138]; Suupohja [141]; Seinäjoki [142]; Kuusiokunnat [144]; Järviseutu [146]; Vaasa [152]; Sydösterbotten [153]; Jakobstadsregionen [154]; Kaustinen [161]; Kokkola [162]; Oulu [171]; Oulunkaari [173]; Raahe [174]; Haapavesi-	

			Siikalatva [175]; Nivala-Haapajärvi [176]; Ylivieska [177]; Koillismaa [178]; Kehys-Kainuu [181]; Kajaani [182]; Rovaniemi [191]; Kemi-Tornio [192]; Torniolaakso [193]; Itä-Lappi [194]; Tunturi-Lappi [196]; Pohjois-Lappi [197]; Mariehamns stad [211]; Ålands landsbygd [212]; Ålands skärgård [213].	
Number of previous childbirth	Covariate for model adjustment	Childbirth prior to start of follow-up.	NA	Medical Birth Register (Finnish Institute for Health and Welfare)
Civil status	Covariate for model adjustment	Civil status denotes an individual's legal standing in relation to others, as defined by family law statutes, including the Marriage Act and the Act on Registered Partnerships.	Unmarried; Married; Divorced; Widowed; Other	Population Register (Statistics Finland)
Socio-economic group	Covariate for model adjustment	General indicator of the social and economic environment.	Self-employed; Upper-level employees; Lower-level employees; Manual workers; Students; Pensioners; Others; Unknown	Population Register (Statistics Finland)
Education	Covariate for model adjustment	Highest level of education based on The International Standard Classification of Education (ISCED) classification.	Upper secondary; Post-secondary non-tertiary, Short-cycle tertiary, Bachelor, Master, Doctoral, Unknown	Population Register (Statistics Finland)

Diabetes (Kela code 103 or 215)	Covariate for model adjustment	Based on Kela reimbursement codes for classification of chronic disease. Entitlement to reimbursement is based on a medical certificate, including one of the ICD-10 codes specified (E10-E14, E89.1), from a physician.	**No**; Yes	Population Register (Statistics Finland)
Hypothyroidism (Kela code 104)	Covariate for model adjustment	Based on Kela reimbursement codes for classification of chronic disease. Entitlement to reimbursement is based on a medical certificate, including one of the ICD-10 codes specified (C73, E03, E89.0), from a physician.	**No**; Yes	Social Insurance Institution of Finland
Breast cancer (Kela code 115)	Covariate for model adjustment	Based on Kela reimbursement codes for classification of chronic disease. Entitlement to reimbursement is based on a medical certificate, including one of the ICD-10 codes specified (C50, D05.1), from a physician.	**No**; Yes	Social Insurance Institution of Finland
Leukaemia, lymphoma, or other malignancy of blood or bone marrow (Kela code 117)	Covariate for model adjustment	Based on Kela reimbursement codes for classification of chronic disease. Entitlement to reimbursement is based on a medical certificate, including one of the ICD-10 codes specified (C81–C85, C88, C90–C96, D45–D47, D72.1, D75, E85), from a physician.	**No**; Yes	Social Insurance Institution of Finland

Gynaecologic cancer (Kela code 128)	Covariate for model adjustment	Covariate for model adjustment. Based on Kela reimbursement codes for classification of chronic disease. Entitlement to reimbursement is based on a medical certificate, including one of the ICD-10 codes specified (D39, C51–C58), from a physician.	**No**; Yes	Social Insurance Institution of Finland
Other malignancy (Kela code 130)	Covariate for model adjustment	Based on Kela reimbursement codes for classification of chronic disease. Entitlement to reimbursement is based on a medical certificate, including one of the ICD-10 codes specified (C00–C26, C30–C34, C37–C41, C43–C49, C60, C62–C80, C97, D33, D43, D48), from a physician.	**No**; Yes	Social Insurance Institution of Finland
Connective tissue disease (Kela code 202)	Covariate for model adjustment	Based on Kela reimbursement codes for classification of chronic disease. Entitlement to reimbursement is based on a medical certificate, including one of the ICD-10 codes specified (A04.6, A39.8, A50.5, D76, H20, H30, I33.0, I40.8, J84, K50.9, K51.9, K73.2, K74.3, K75.4, K83.0, L40.5, M02, M05, M06, M08, M13, M30–M35, M45, M46.1, M46.9, M86.3, M86.6, M94.1, N03, N04, Q44.2), from a physician.	**No**; Yes	Social Insurance Institution of Finland
Inflammatory bowel disease	Covariate for model	Based on Kela reimbursement codes for classification of	**No**; Yes	Social Insurance Institution of Finland

(Kela code 208)	adjustment	chronic disease. Entitlement to reimbursement is based on a medical certificate, including one of the ICD-10 codes specified (K50, K51), from a physician.		
Obesity	Covariate for model adjustment	Diagnosis of obesity prior to start of follow-up, based on ICD-10 code E66.	**No**; Yes	Care Register of Primary Health Care Visits (Finnish Institute for Health and Welfare) and Care Register for Health Care (Finnish Institute for Health and Welfare)
Thrombosis, embolism or related endpoint event	Covariate for model adjustment	Diagnosis of thromboembolic event prior to start of follow-up based on ICD-10 codes (I80, I81, I82, I21, I26, I63).	**No**; Yes	Care Register of Primary Health Care Visits (Finnish Institute for Health and Welfare) and Care Register for Health Care (Finnish Institute for Health and Welfare)
Heavy menstrual bleeding	Covariate for model adjustment	Diagnosis of heavy menstrual bleeding or related condition prior to start of follow-up based on ICD-10 codes (N80, N84, N85, N92, N93, D25).	**No**; Yes	Care Register of Primary Health Care Visits (Finnish Institute for Health and Welfare) and Care Register for Health Care (Finnish Institute for Health and Welfare)
Use of hormonal contraception during follow-up (individual product)	Exposure	Short-acting reversible contraception use was defined as having at least two consecutive redeemed prescriptions of the same ATC code in the year (360 days) prior to the failure event. For long-acting reversible contraception users (LNG-IUDs and contraceptive implants), only one redeemed prescription in the five years (1800	**No hormonal contraception**; Levonorgestrel and ethinylestradiol (ATC G03AA07)); Desogestrel and ethinylestradiol (ATC G03AA09); Gestodene and ethinylestradiol (ATC G03AA10); Drospirenone and ethinylestradiol (ATC G03AA12); Dienogest and	Prescription Centre in the Kanta Services. Hormonal contraception use was defined using the Anatomical Therapeutic Chemical (ATC) codes for G02B (contraceptives for topical use), G03A (hormonal contraceptives for systematic use) or G03HB (antiandrogens and oestrogens). Emergency

		days) prior to failure event was required.	ethinylestradiol (ATC G03AA16); Levonorgestrel and ethinylestradiol, sequential (ATC G03AB03); Cyproterone and ethinylestradiol (ATC G03HB01); Nomegestrol and oestradiol (ATC G03AA14); Dienogest and oestradiol (G03AB08); Norethisterone (ATC G03AC01); Desogestrel (ATC G03AC09); Drospirenone (ATC G03AC10); Vaginal ring with progestogen and ethinylestradiol (ATC G02BB01); Contraceptive patch with norelgestromin and ethinylestradiol (ATC G03AA13); Plastic IUD with levonorgestrel, i.e., LNG-IUD (ATC G02BA03); Contraceptive implant with levonorgestrel (ATC G03AC03); Contraceptive implant with etonogestrel (ATC G03AC08)	contraceptives (ATC code G03AD) are widely available over-the-counter in Finland and therefore excluded from this study.
Use of hormonal contraception group during follow-up (product group)	Exposure	Categorial variable grouping based on hormonal content and mode of delivery. Short-acting reversible contraception use was defined as having at	**No hormonal contraception**; Combined oral contraceptives, EE (ethinylestradiol) *[levonorgestrel and*	Prescription Centre in the Kanta Services, grouping of individual products. Hormonal contraception use was defined using the Anatomical

		least two consecutive redeemed prescriptions of the same ATC code in the year (360 days) prior to the failure event. For long-acting reversible contraception users (LNG-IUDs and contraceptive implants), only one redeemed prescription in the five years (1800 days) prior to failure event was required.	*ethinylestradiol, desogestrel and ethinylestradiol, gestodene and ethinylestradiol, drospirenone and ethinylestradiol, dienogest and ethinylestradiol, levonorgestrel and ethinylestradiol (sequential), cyproterone and ethinylestradiol]*; Combined oral contraceptives, E2 (natural oestradiol) *[nomegestrol and oestradiol, dienogest and oestradiol]*; Progestin-only oral contraceptives *[norethisterone, desogestrel, drospirenone]*; Vaginal contraceptive rings *[vaginal ring with progestogen and ethinylestradiol]*; Contraceptive patches *[norelgestromin and ethinylestradiol]*; Hormonal intrauterine devices *[LNG-IUD]*; Contraceptive implants *[contraceptive implant with levonorgestrel, contraceptive implant with etonogestrel]*	Therapeutic Chemical (ATC) codes for G02B (contraceptives for topical use), G03A (hormonal contraceptives for systematic use) or G03HB (antiandrogens and oestrogens). Emergency contraceptives (ATC code G03AD) are widely available over-the-counter in Finland and therefore excluded from this study.

Table S2: Nested case-control results. Odds ratios for crude model (OR) and adjusted model (aOR), along with their respective upper (OR.hi, aOR.hi) and lower (OR.lo, aOR.lo) bounds within a 95% confidence interval. COC = combined oral contraceptive, EE = ethinylestradiol, E2 = oestrogen, IUD = intrauterine device.

HC use	Control (n)	Case (n)	OR	OR.hi	OR.lo	aOR	aOR.hi	aOR.lo
GROUP: NO HORMONAL CONTRACEPTION	8752	2384	1.00	1.00	1.00	1.00	1.00	1.00
GROUP: COC, EE	2264	438	0.70	0.78	0.62	0.76	0.86	0.68
Levonorgestrel/ EE (fixed)	59	8	0.50	1.04	0.24	0.54	1.14	0.26
Desogestrel/ EE (fixed)	266	62	0.82	1.09	0.62	0.87	1.16	0.65
Gestodene/ EE (fixed)	490	65	0.48	0.63	0.37	0.55	0.72	0.42
Drospirenone/ EE (fixed)	1067	212	0.72	0.84	0.61	0.78	0.91	0.66
Dienogest/ EE (fixed)	85	19	0.79	1.32	0.48	0.84	1.40	0.50
Levonorgestrel/ EE (sequential)	<5	<5	2.19	24.25	0.20	1.93	24.71	0.15
Cyproterone/ EE (fixed)	295	71	0.87	1.13	0.66	0.96	1.26	0.73
VAGINAL RING: Etonogestrel/EE	264	48	0.63	0.87	0.46	0.70	0.97	0.51
CONTRACEPTIVE PATCH: Norelgestromin/ EE	33	8	0.78	1.71	0.36	0.74	1.67	0.33
GROUP: COC, E2	335	41	0.47	0.65	0.34	0.49	0.68	0.35
Nomegestrol/ E2 (fixed)	160	18	0.41	0.68	0.25	0.44	0.73	0.27
Dienogest/ E2 (sequential)	175	23	0.52	0.81	0.34	0.55	0.85	0.35
GROUP: PROGESTIN-ONLY ORAL CONTRACEPTIVES	1202	130	0.41	0.49	0.34	0.42	0.51	0.35
Norethisterone	86	15	0.62	1.08	0.36	0.60	1.07	0.34
Desogestrel	1106	111	0.38	0.46	0.31	0.40	0.48	0.32
Drospirenone	10	<5	1.44	4.62	0.45	1.51	5.04	0.45
PLASTIC IUD: Levonorgestrel	187	32	0.63	0.93	0.43	0.62	0.92	0.42
GROUP: CONTRACEPTIVE IMPLANT	106	19	0.64	1.05	0.39	0.57	0.96	0.34
Etonorgestrel	41	10	0.86	1.73	0.42	0.70	1.46	0.34

Levonorgestrel	65	9	0.50	1.02	0.25	0.49	1.01	0.23

Figure S1: Mindmap of factors related to hormonal contraception and anaemia used for creating the directed acyclic graph. Inclusion of factors was based on previous literature and FinRegistry correlations of likely associations for iron deficiency anaemia in the Finnish population.

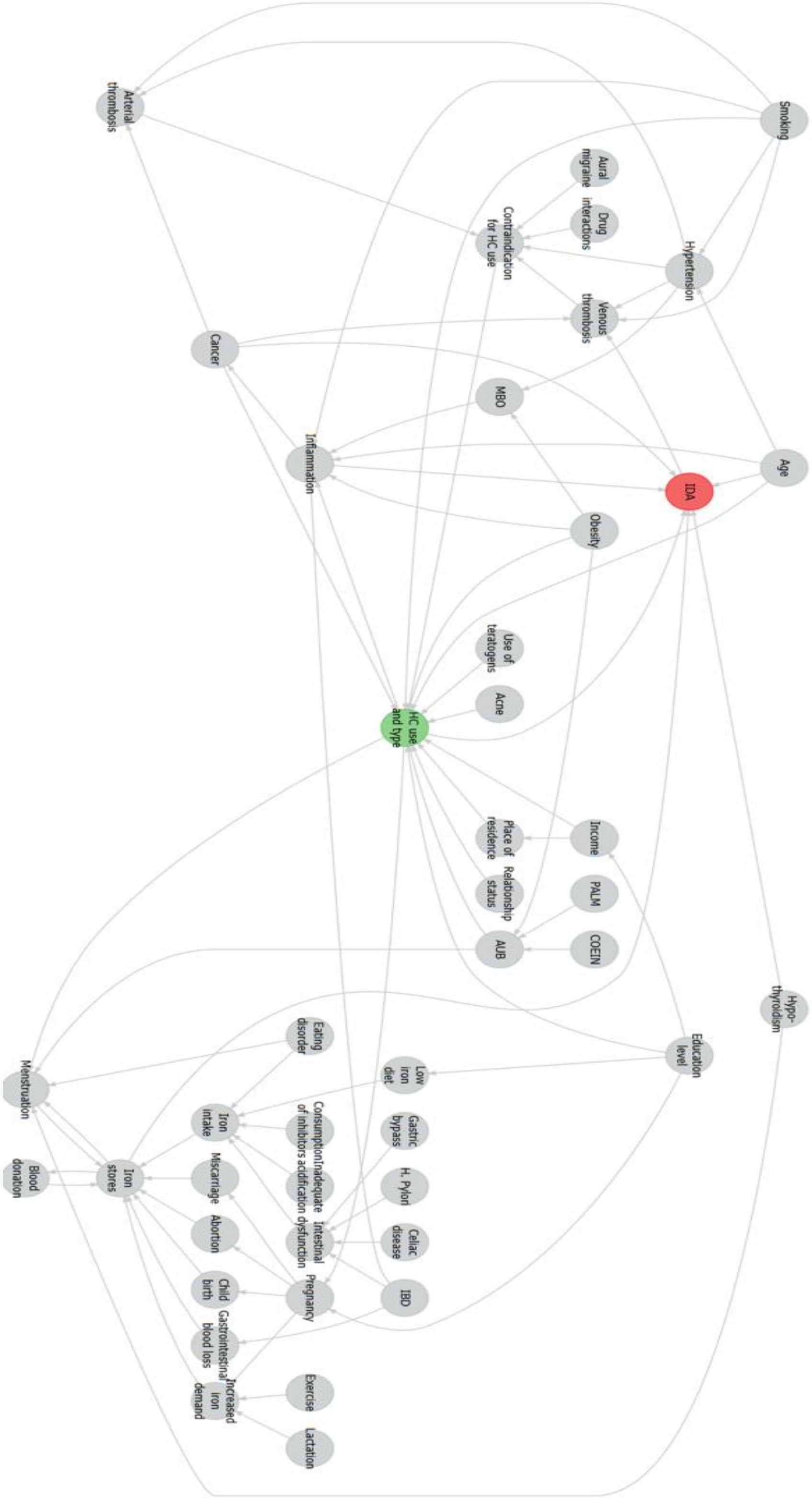

Figure S2: Frequency distribution of hormonal contraceptive use and non-use by age in cases and controls for products with ≥20 users. The number within the grid represents the number of cases and controls within each group. The vertical red line represents the mean age of users and non-users of the specified hormonal contraception (no hormonal contraceptive users 33·75 years; levonorgestrel and ethinylestradiol (fixed preparations) 29·16 years; desogestrel and ethinylestradiol 30·32 years; gestodene and ethinylestradiol 29·92 years; drospirenone and ethinylestradiol 27·77 years; dienogest and ethinylestradiol 25·17 years; cyproterone and ethinylestradiol 30·54 years; nomegestrol and oestradiol 31·24 years, dienogest and oestradiol 38·87 years, norethisterone-only 33·38 years, desogestrel-only 35·79 years; vaginal ring releasing etonogestrel and ethinylestradiol 32·26 years; contraceptive patch releasing norelgestromin and ethinylestradiol 33·34 years; levonorgestrel-releasing IUD 36·10 years; etonogestrel-releasing contraceptive implant 29·60 years; levonorgestrel-releasing contraceptive implant 35·00 years). OC = oral contraceptive. IUD = intrauterine device.

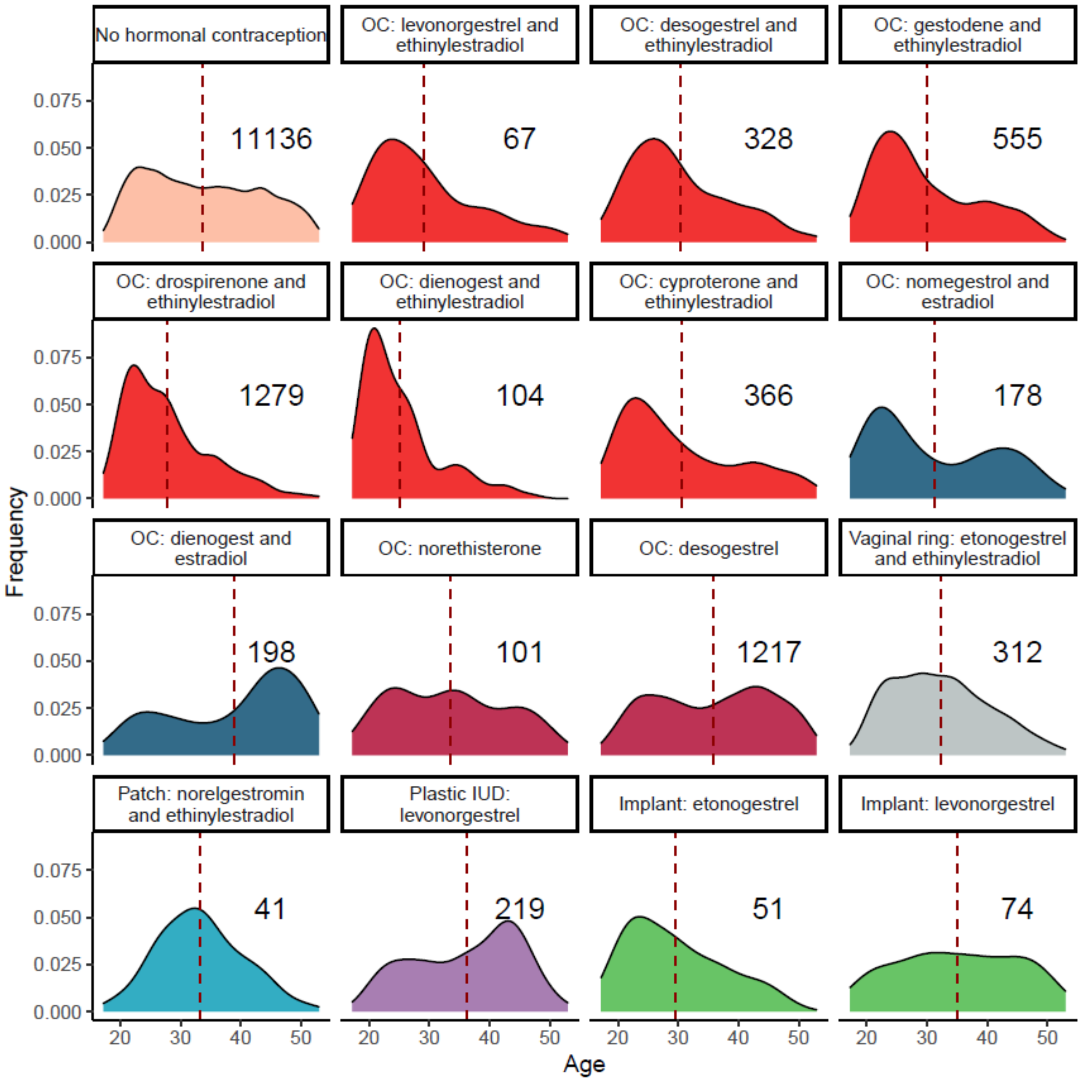

Table S3: Nested case-control results for fully adjusted model and different time windows of hormonal contraception exposure. Panel A shows NCC results of the fully adjusted model accounting for civil status, socioeconomic group, highest level of education, history of childbirth, chronic disease (diabetes, hypothyroidism, cancer, connective tissue disease, inflammatory bowel disease, thrombosis and related events, and obesity), and heavy menstrual bleeding. Panel B shows nested case control study results using 3-month time window, and Panel C using a 6-month time window. Odds ratios (OR) are presented along with their respective upper (OR.hi) and lower (OR.lo) bounds within a 95% confidence interval. COC = combined oral contraceptive, EE = ethinylestradiol, E2 = oestrogen, IUD = intrauterine device.

	Panel A: Fully adjusted model (considering socioeconomic factors and chronic disease)					Panel B: Unadjusted model; 3 month (90 days) time window					Panel C: Unadjusted model; 6 month (180 days) time window				
	Control (n)	Case (n)	OR	OR. hi	OR. lo	Control (n)	Case (n)	OR	OR. hi	OR. lo	Control (n)	Case (n)	OR	OR. hi	OR. lo
GROUP: NO HORMONAL CONTRACEPTION	8752	2384	1.00	1.00	1.00	10181	2646	1.00	1.00	1.00	10384	2709	1.000	1.000	1.000
GROUP: COC, EE	2264	438	0.76	0.67	0.85	2135	420	0.74	0.66	0.83	1888	355	0.71	0.63	0.80
Levonorgestrel/ EE (fixed)	59	8	0.54	0.26	1.13	59	12	0.78	0.42	1.45	53	10	0.708	0.359	1.396
Desogestrel/ EE (fixed)	266	62	0.88	0.66	1.17	252	53	0.78	0.57	1.05	207	42	0.744	0.532	1.042
Gestodene/ EE (fixed)	490	65	0.53	0.41	0.70	433	63	0.55	0.42	0.72	402	51	0.480	0.357	0.645
Drospirenone/ EE (fixed)	1067	212	0.77	0.66	0.90	994	191	0.73	0.62	0.86	861	163	0.720	0.604	0.859
Dienogest/ EE (fixed)	85	19	0.87	0.52	1.45	98	20	0.75	0.46	1.22	83	17	0.764	0.450	1.297
Levonorgestrel/ EE (sequential)	<5	<5	2.53	0.23	28.08	31	9	1.00	0.47	2.11	25	7	0.953	0.409	2.220
Cyproterone/ EE (fixed)	295	71	0.94	0.72	1.23	268	72	1.03	0.79	1.34	257	65	0.961	0.728	1.268
VAGINAL RING: Etonogestrel/ EE	264	48	0.70	0.51	0.96	232	41	0.65	0.46	0.91	225	36	0.59	0.41	0.84
CONTRACEPTIVE PATCH: Norelgestromin/ EE	33	8	0.79	0.35	1.77	<5	<5	NA	NA	NA	<5	<5	NA	NA	NA
GROUP: COC, E2	335	41	0.49	0.35	0.68	316	42	0.53	0.38	0.74	276	37	0.54	0.38	0.76
Nomegestrol/ E2 (fixed)	160	18	0.44	0.27	0.72	134	17	0.49	0.29	0.81	119	15	0.480	0.279	0.826
Dienogest/ E2 (sequential)	175	23	0.53	0.34	0.83	182	25	0.57	0.37	0.87	157	22	0.583	0.371	0.914
GROUP: PROGESTIN-ONLY ORAL CONTRACEPTIVES	1202	130	0.41	0.34	0.50	1186	145	0.48	0.40	0.57	1021	102	0.39	0.32	0.48
Norethisterone	86	15	0.65	0.37	1.14	81	11	0.50	0.27	0.95	74	10	0.498	0.256	0.968
Desogestrel	1106	111	0.38	0.31	0.47	1076	120	0.44	0.36	0.53	929	85	0.362	0.289	0.454
Drospirenone	10	<5	1.65	0.50	5.37	29	14	1.84	0.97	3.50	18	7	1.448	0.601	3.485
PLASTIC IUD: Levonorgestrel	187	32	0.63	0.43	0.93	56	15	1.08	0.61	1.91	116	30	0.99	0.66	1.49
GROUP: CONTRACEPTIVE IMPLANT	106	19	0.57	0.34	0.96	9	6	2.56	0.91	7.22	74	17	0.87	0.51	1.48

Etonorgestrel	41	10	0.71	0.34	1.49	9	6	2.56	0.91	7.22	16	8	1.914	0.815	4.492
Levonorgestrel	65	9	0.48	0.23	0.99	<5	<5	NA	NA	NA	58	9	0.571	0.279	1.166

Table S4: Sensitivity analysis of nested case control study. Odds ratios (OR) are presented along with their respective upper (OR.hi) and lower (OR.lo) bounds within a 95% confidence interval. COC = combined oral contraceptive, EE = ethinylestradiol, E2 = oestrogen, IUD = intrauterine device.

	Model S1: Number of previous previous pregnancy					Model S2: women younger than 25 years old excluded					Model S3: pensioners excluded				
	Control (n)	Case (n)	OR	OR. hi	OR. lo	Control (n)	Case (n)	OR	OR. hi	OR. lo	Control (n)	Case (n)	OR	OR. hi	OR. lo
GROUP: NO HORMONAL CONTRACEPTION	8752	2384	1	1	1	6663	1872	1	1	1	7717	2110	1	1	1
GROUP: COC, EE	2264	438	0.77	0.86	0.68	1376	237	0.64	0.74	0.55	2067	395	0.72	0.82	0.64
Levonorgestrel/ EE (fixed)	59	8	0.55	1.15	0.26	35	6	0.62	1.48	0.26	51	6	0.43	1.01	0.18
Desogestrel/ EE (fixed)	266	62	0.88	1.17	0.66	189	41	0.75	1.06	0.53	245	54	0.79	1.08	0.59
Gestodene/ EE (fixed)	490	65	0.55	0.72	0.42	318	36	0.44	0.63	0.31	434	60	0.54	0.71	0.41
Drospirenone/ EE (fixed)	1067	212	0.78	0.92	0.67	612	108	0.66	0.82	0.53	990	193	0.75	0.89	0.63
Dienogest/ EE (fixed)	85	19	0.84	1.40	0.51	32	9	0.90	1.91	0.42	80	17	0.73	1.25	0.42
Levonorgestrel/ EE (sequential)	<5	<5	1.91	24.49	0.15	<5	<5	1.75	21.55	0.14	<5	<5	1.79	22.30	0.14
Cyproterone/ EE (fixed)	295	71	0.97	1.27	0.74	188	36	0.71	1.02	0.49	265	64	0.92	1.22	0.69
VAGINAL RING: Etonogestrel/ EE	264	48	0.71	0.98	0.51	201	41	0.75	1.07	0.53	237	42	0.66	0.93	0.47
CONTRACEPTIVE PATCH: Norelgestromin/ EE	33	8	0.73	1.65	0.32	32	6	0.55	1.35	0.22	32	7	0.70	1.61	0.30
GROUP: COC, E2	335	41	0.50	0.70	0.36	244	29	0.48	0.71	0.32	287	39	0.54	0.76	0.38
Nomegestrol/ E2 (fixed)	160	18	0.44	0.73	0.27	97	10	0.39	0.76	0.20	138	16	0.42	0.71	0.25
Dienogest/ E2 (sequential)	175	23	0.55	0.85	0.35	147	19	0.54	0.88	0.33	149	23	0.67	1.04	0.42
GROUP: PROGESTIN-ONLY ORAL CONTRACEPTIVES	1202	130	0.42	0.51	0.35	976	109	0.43	0.53	0.35	1031	119	0.44	0.54	0.36
Norethisterone	86	15	0.60	1.07	0.34	64	12	0.62	1.17	0.33	69	13	0.65	1.19	0.36
Desogestrel	1106	111	0.40	0.49	0.32	904	94	0.41	0.51	0.32	956	102	0.42	0.51	0.34
Drospirenone	10	<5	1.50	5.01	0.45	8	<5	1.22	4.94	0.30	6	<5	2.20	8.32	0.58
PLASTIC IUD: Levonorgestrel	187	32	0.62	0.91	0.42	163	24	0.54	0.84	0.35	167	30	0.67	1.00	0.45
GROUP: CONTRACEPTIVE IMPLANT	106	19	0.58	0.97	0.34	78	14	0.59	1.06	0.32	90	16	0.62	1.08	0.36
Etonorgestrel	41	10	0.70	1.46	0.33	26	6	0.63	1.61	0.25	35	9	0.84	1.79	0.40
Levonorgestrel	65	9	0.49	1.02	0.24	52	8	0.56	1.22	0.26	55	7	0.47	1.05	0.21
